# Human amniotic membrane application in oral surgery—An *ex vivo* pilot study

**DOI:** 10.3389/fbioe.2022.968346

**Published:** 2022-10-24

**Authors:** Stéphane Odet, Lauriana Solecki, Christophe Meyer, Elise Weber, Brice Chatelain, Edouard Euvrard, Aude Barrabé, Thomas Gualdi, Anne-Laure Parmentier, Laurent Tatu, Fabienne Pouthier, Aurélien Louvrier, Florelle Gindraux

**Affiliations:** ^1^ Service de chirurgie maxillo-faciale, stomatologie et odontologie hospitaliéère, CHU Besançon, Besançon, France; ^2^ Service d’ophtalmologie, CHU Besançon, Besançon, France; ^3^ Laboratoire de Nanomédecine, Imagerie, Thérapeutique EA 4662, Université Bourgogne Franche-Comté, Besançon, France; ^4^ INSERM Centre d’Investigation Clinique 1431, CHU Besançon, Besançon, France; ^5^ Service de neurologie, CHU Besançon, Besançon, France; ^6^ Laboratoire d’anatomie, Université Bourgogne Franche-Comté, Besançon, France; ^7^ AICT, Établissement français du sang Bourgogne Franche-Comté, Besançon, France; ^8^ Université Bourgogne Franche-Comté, INSERM, EFS BFC, UMR1098, RIGHT Interactions Greffon-Hôte-Tumeur/Ingénierie Cellulaire et Génique, Besançon, France

**Keywords:** amniotic membrane, porcine jaw, oral mucosa, xenograft, *ex vivo*, osteonecrosis of the jaw and illustrations

## Abstract

**Objectives:** The purpose of this pilot porcine study was to explore and illustrate the surgical application of human amniotic membrane (hAM) in an *ex vivo* model of medication-related osteonecrosis of the jaw (MRONJ).

**Material and methods:** Five oral and maxillofacial surgeons participated to this study. MRONJ was simulated on porcine mandible specimens. hAM was applied using four different techniques: implantation with complete coverage, implantation with partial coverage, apposition and covering graft material. At the same time, the surgeons evaluated how well the hAM handled and its physical properties during the surgery.

**Results:** Surgeons found that hAM had suitable mechanical properties, as it was easy to detach from the support, handle, bind to the defect and bury. hAM was also found to be strong and stable. The “implantation with complete coverage” and “implantation with partial coverage” techniques were the preferred choices for the MRONJ indication.

**Conclusion:** This study shows that hAM is a graft material with suitable properties for oral surgery. It is preferable to use it buried under the gingiva with sutures above it, which increases its stability. This technical note aims to educate surgeons and provide them with details about the handling of hAM in oral surgery.

**Clinical relevance:** Two surgical techniques for hAM application in MRONJ were identified and illustrated. hAM handling and physical properties during surgery were reported.

## Introduction

Human amniotic membrane (hAM) is the innermost layer of fetal membranes. It is composed of a single layer of epithelial cells, a basement membrane, and an avascular stroma containing amniotic mesenchymal stem cells, underlayered by the chorion. Its thickness (70–180 µm) varies among individuals (Chen et al., 2012; Gremare et al., 2019). The beneficial effects of hAM use have been widely described in the literature. To date, ophthalmology is one of the most popular applications of hAM (John, 2003).

Since the mid-1990s, there has been a growing interest in using hAM for oral surgery to accelerate tissue regeneration. One systematic review of literature explored the different indications for hAM use in oral surgery (Fenelon et al., 2018). In this line, two hAM configurations were identified (Odet et al., 2021): “implanted graft material” and “covering graft material”. The first one was applied to gingival recession, bone defects in the furcation, bone defects in interproximal areas and surgical wounds after implant surgery. The second one was applied to mandibular vestibuloplasty and mucosal defects. Whereas hAM use in ophthalmology has been accompanied by informative surgical illustrations (Dua and Azuara-Blanco, 1999; Letko et al., 2001; John, 2003), its use in oral surgery is not described to the same extent, specifically its handling and surgical application. As a consequence, a specific nomenclature beyond the previously mentioned terms—“implanted graft material” and “covering graft material”—was necessary (Odet et al., 2021). Along these lines, four theorical types of hAM surgeries are proposed:

1) *“implantation”*, where the hAM is buried and completely covered by the gingiva
2) *“apposition”*, where the hAM is applied against the site to be treated, not sutured, left exposed in the mouth and stabilized by any means (cross stitches, pressure dressing, palatal plates, etc.)
3) *“whole covering graft material”*, where the hAM is applied against the site to be treated, sutured to adjacent mucosa or underlying mucosa, fully left exposed in the mouth and protected by any means (cross stitches, pressure dressing, palatal plates, etc.)
4) *“partial covering graft material”*, where the hAM is applied against the bone, buried under the wound edges, sutured to adjacent mucosa or underlying mucosa, left partially exposed in the mouth and protected by any means (cross stitches, pressure dressing, palatal plates, etc.).

However, no study has provided details about how to handle cryopreserved hAM which is more challenging to cut, orient (mesenchymal versus epithelial side), manipulate and apply than the lyophilized or dehydrated amnion or amnion-chorion often used in oral surgery (Fenelon et al., 2018; Gulameabasse et al., 2020; Odet et al., 2021).

As previously investigated by Ragazzo et al. (Ragazzo et al., 2018; Ragazzo et al., 2021), our team wanted to use hAM to manage medication-related osteonecrosis of the jaw (MRONJ) in a compassionate clinical trial (Odet et al., 2022). Despite our extensive experience with hAM banking, its *in vitro*/*in vivo* osteogenic potential, and its use in oral, bone and nerve surgeries (Gindraux and Obert, 2010; Obert et al., 2012; Gindraux et al., 2013; Laurent et al., 2014a; Laurent et al., 2014b; Laurent et al., 2014c; Gindraux et al., 2017; Laurent et al., 2017; Fénelon et al., 2018; Bourgeois et al., 2019; Fenelon et al., 2019; Gualdi et al., 2019; Fenelon et al., 2020; Etchebarne et al., 2021; Fénelon et al., 2021; Fenelon et al., 2021; Odet et al., 2021), we failed to identify how to handle and apply hAM during surgery in the oral cavity. Thus, an *ex vivo* pilot study was required to fill these voids and train the surgeons.

Porcine jaw specimens are common *in vivo* models for oral and maxillofacial surgery, as the bone, teeth and mucosa have similar appearance, size and structure as the human jaw (Deppe et al., 2018; Kniha et al., 2021). So, MRONJ was simulated in fresh porcine mandible specimens to investigate 1) the handling of cryopreserved hAM and its related physical properties for oral surgery, and 2) the four previously listed theorical types of hAM surgeries. No specific MRONJ grade was targeted in this study. A questionnaire was developed to collect surgeon feedback. Thus, this technical note only defines hAM handling, its physical properties and surgical application in an *ex vivo* MRONJ model. It provides surgeons with tips and tricks for hAM application in oral surgery or more broadly, in soft tissue regeneration.

## Material and methods

The work for this technical note was performed at the anatomy laboratory of the University of Franche-Comté (Besançon, France). Five maxillofacial and oral surgeons (CM, EE, EW, AB, SO), one ophthalmologist and one methodologist participated in the training. The fresh porcine mandible specimens were provided by Chevillotte Breeders (Valdahon, France). All the hAM application techniques were filmed by the group “Tête de Com”, and all the illustrations were made by Mr. Thomas Gualdi, a scientific illustrator.

hAM suitable for scientific purposes were provided by the AICT bank from the French Blood Institute (Etablissement Français du Sang). A 4.7-cm diameter cryopreserved hAM stored in glycerol on a nitrocellulose support (epithelial layer facing the support) was thawed for 2 h at room temperature. After three 5 min rinses in saline or hypotonic injection solution, the hAM was cut either on the nitrocellulose support or after being detached from it.

### MRONJ simulation

**FIGURE 1:**
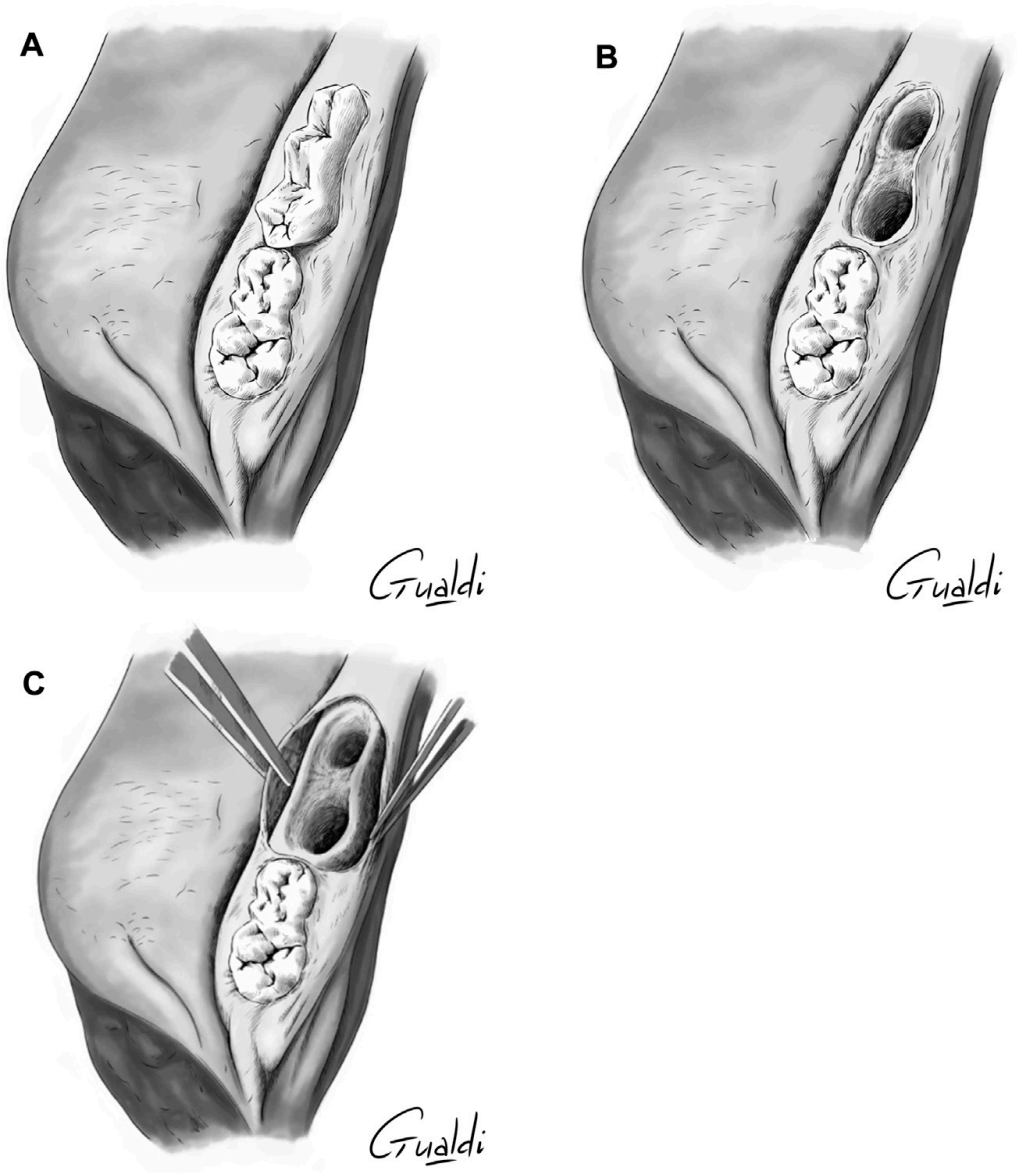
Simulation of MRONJ on porcine mandible specimen (upper view illustration): **(A)** Before premolar extraction; **(B)** After premolar extraction; **(C)** After gingival detachment from the alveolar bone.

A sulcular incision on two adjacent teeth was made in the premolar area of the porcine mandible (Figures 1A–C). Two teeth were extracted, and a 3-mm wide defect was created by resecting the mucosa on the vestibular edge of the incision, simulating MRONJ. The alveolar bone was resected over approximately 3 mm using a rongeur. Here, no specific MRONJ grade was targeted.

For the “hAM implantation with complete coverage” technique (see below), a horizontal periosteum incision was made on the two full thickness vestibular and lingual flaps to allow tensionless closure on the mucosal edges.

### hAM applications

**FIGURE 2:**
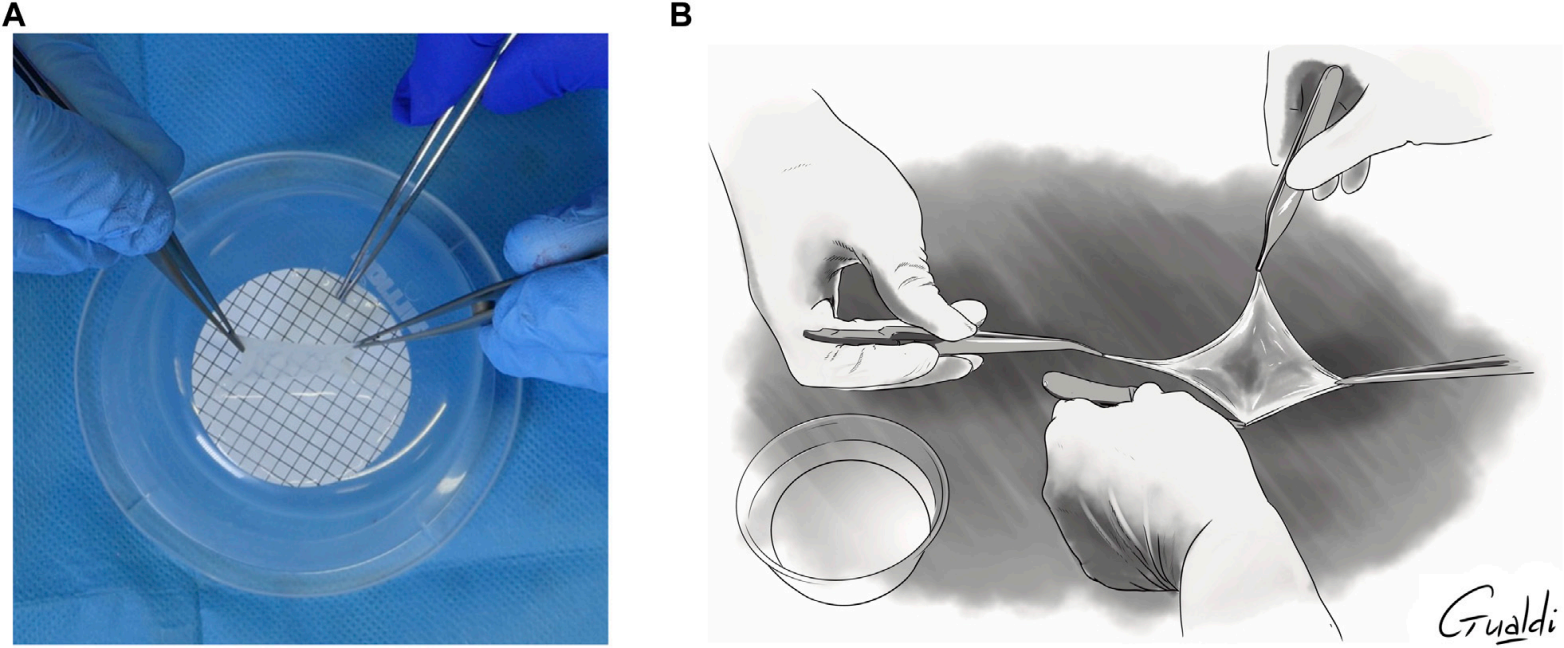
**(A)** hAM detachment from the nitrocellulose support. **(B)** “Four hands” application of hAM.

Two surgeons were needed to separate the hAM from its support: one detached the hAM with two forceps (without teeth) while the other held the support with another set of forceps (Figure 2A). The hAM tended to fold upon itself once detached from the support. Two options were used to unfold and apply it. The first one needed two surgeons: one held the membrane while the second unfolded it using two forceps without teeth. Later, both surgeons applied it on the surgical site (“with four hands”) in the desired orientation (Figure 2B). In the second option, only one surgeon was required: once the hAM was detached from the support, it was directly applied on the surgical site, and then unfolded using two forceps. The hAM’s orientation was quite difficult to maintain in this case.

**FIGURE 3:**
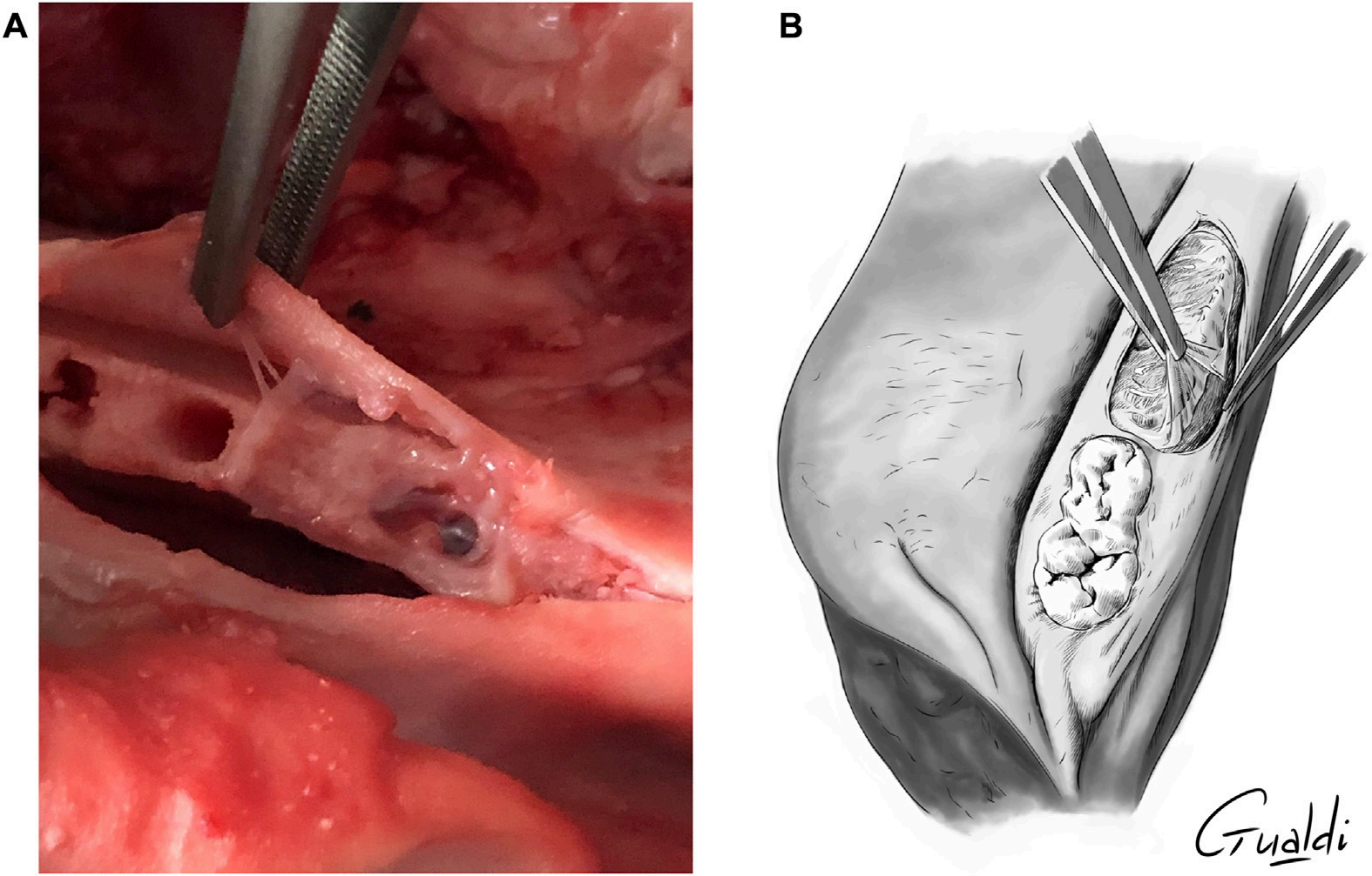
**(A)** hAM application on MRONJ simulation site. **(B)** hAM burying between bone and gingiva (upper view illustration).

The four theoretical techniques identified by Odet et al. (Odet et al., 2021) were attempted and adapted to this experimental study. After hAM application at the MRONJ site (Figure 3A), hAM was buried between bone and gingiva when necessary (Figure 3B). In all cases, hAM could be cut into the desired shape and size and then applied with the mesenchymal side facing the bone and the epithelial side facing the gingiva. One to two hAM units were manipulated by the surgeon.

### Evaluation by surgeons

hAM handling and physical properties during the surgery were evaluated with a questionnaire. The studied parameters were:

1) ease of detaching the hAM from the nitrocellulose support
2) hAM handling
3) hAM adhesion once applied on the defect (bone)
4) hAM strength
5) ease of suturing the hAM
6) ease of burying the hAM between the bone and mucosa.

These parameters were evaluated on a scale of 0–10 (0 = impossible to handle the hAM/failure of the procedure; 10 = perfect handling/success of the procedure). Grading was left up to each surgeon.

Additional parameters included

1) easiest way to cut the hAM: when still bound to the support or after being detached
2) easiest way to apply the hAM on the surgical site: flat or folded on itself
3) stability of hAM during suturing once applied on the surgical site.

## Results

### hAM applications

**FIGURE 4:**
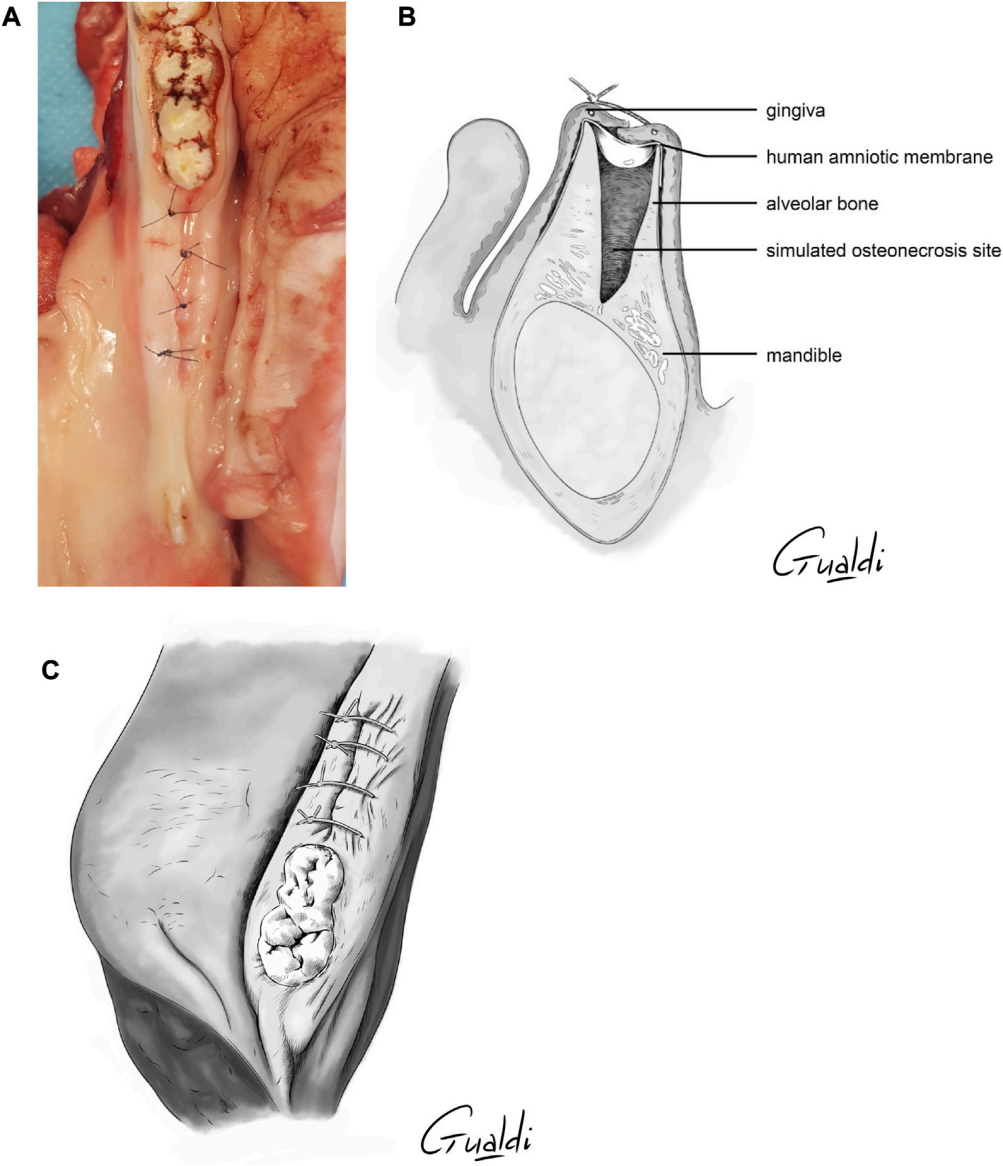
hAM implantation with complete coverage. The sutures were realized above the implanted hAM which was thus not visible. **(A)** Photography; **(B)** Sagittal section illustration; **(C)** Upper view illustration.

**FIGURE 5:**
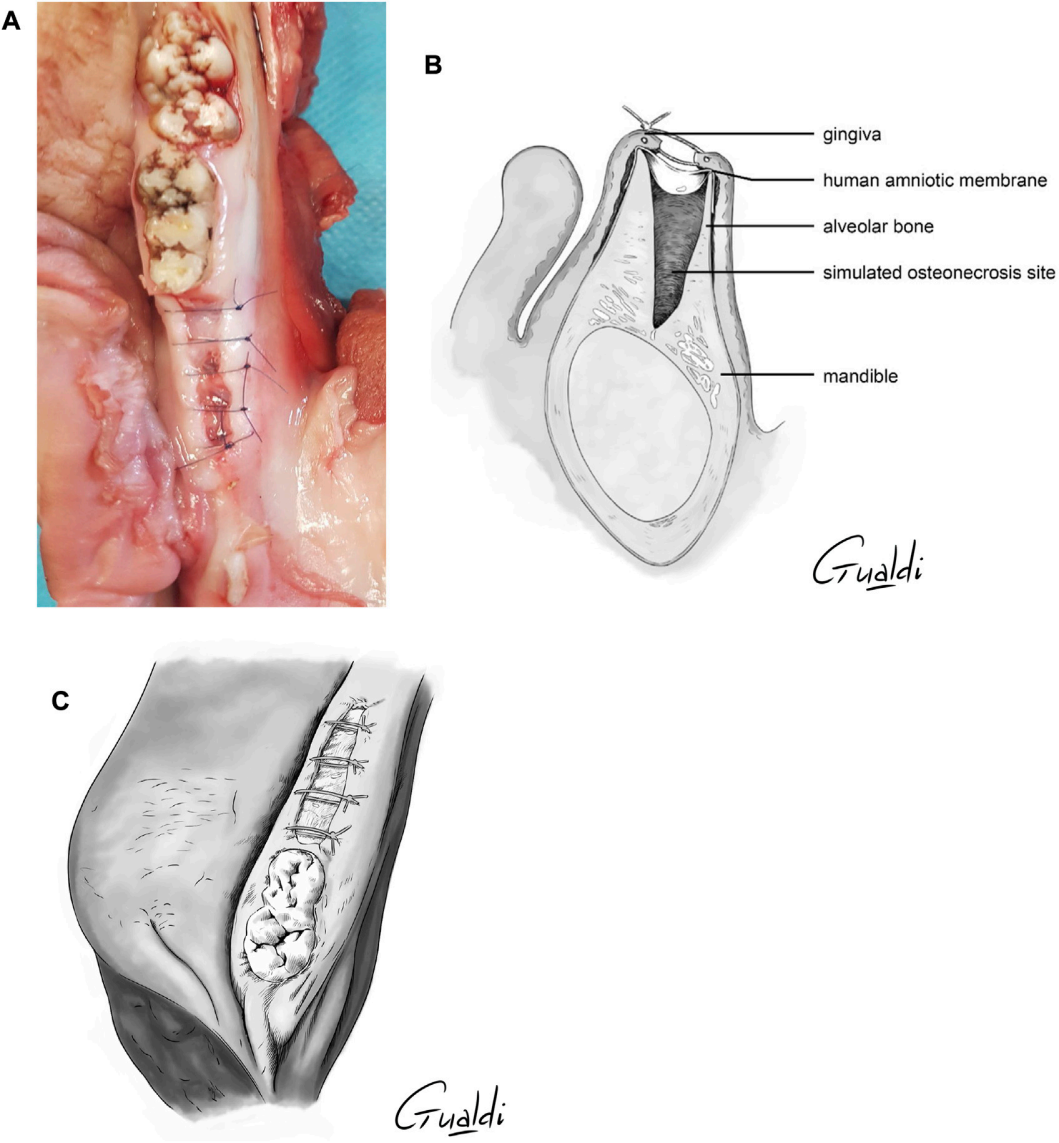
hAM implantation with partial coverage. The gingiva was sutured above the hAM, but leaving the hAM exposed in the oral cavity. **(A)** Photography; **(B)** Sagittal section illustration; **(C)** Upper view illustration.

The four theoretical techniques previously mentioned were attempted and adapted to this practical study as follows:

1) *Implantation with complete coverage* (Figures 4A–C): the hAM was applied and buried between the bone and mucosa (Figure 3B). The mucoperiosteal flap was reapplied over the hAM and sutured hermetically above it, using simple or cross stitches.
2) *Implantation with partial coverage* (Figures 5A–C): the hAM was applied and buried between the bone and mucosa (Figure 3B). The mucoperiosteal flap was then sutured above it, non-hermetically. In this case, the hAM was left exposed in the oral cavity.
3) *Apposition*: the hAM was simply applied “in apposition” against the defect, without burying it between the bone and mucosa. The mucosa was then closed above it as hermetically as possible, using simple or cross stitches. Compared to the initial nomenclature, the hAM was not sutured.
4) *Covering graft material*: The hAM was cut into the desired shape, applied on the defect and sutured directly to the adjacent mucosa, using single stitches.

### Video of the procedure:

https://https://youtu.be/GKy3I-n3NRQ

### Surgeon evaluation

The surgeons noted that hAM was easy to work with overall (Table 1).

1) To detach from the nitrocellulose support: mean of 7.4 (6–8)
2) To handle: mean of 7.0 (4–8)
3) To bind to the defect: mean of 7.4 (6–9)
4) To bury between the bone and mucosa once applied on the defect: mean of 7.6 (5–9).

**TABLE 1:** Evaluation by the surgeons. ND: not done.

Operators	Easiest localization to cut the hAM	Facility to detach hAM from nitrocellulose support	hAM handling	hAM adhesion once applied on the defect	hAM resistance	Facility to suture the hAM	Facility to bury hAM between the bone and the mucosa	Easiest way to apply hAM on the surgical site	Stability of hAM during sutures
**CM**	Support	7	4	6	3	0	5	Folded on itself	YES
**EE**	Support	6	7	9	8	ND	7	Folded on itself	YES
**EW**	Support	8	8	7	10	5	9	Flat	NO
**AB**	Support	8	8	7	10	5	9	Flat	NO
**SO**	Support	8	8	8	9	4	8	Flat	NO
**MEAN**	-	7.4	7	7.4	8	3.5	7.6	-	-

With a mean of 8 (3–10), surgeons found the hAM was very strong during manipulation, particularly when detached from the nitrocellulose support. However, all surgeons had difficulties when suturing it due to its fragility (because the stitch caused a crack during tightening) and its tendency to fold upon itself, making it hard to suture. After thawing and rinsing, wet hAM tended to fold on itself, making it difficult to manipulate and cut it from its nitrocellulose support. All surgeons agreed that it was easier to cut hAM when it was still bound to its support.

Two surgeons mentioned that hAM was easier to apply on the surgical site when it was folded upon itself. They noticed that the folding increased its thickness but that it was impossible to maintain its orientation. The other surgeons found that it was easier to use it flat and mentioned two advantages: hAM orientation and burying between the bone and mucosa.

hAM was found to be unstable at the surgical site during suturing by three surgeons. In these cases, hAM tended to rise up between the stitches when the mucosal edges were approximated. However, it was stable enough that it was not expelled from the surgical site. In contrast, two surgeons found that hAM was quite stable during suturing, without any movement or oral exposition from the hAM.

## Discussion

The aim of this pilot porcine study was to reproduce MRONJ in fresh porcine mandible specimens and to describe hAM handling and physical properties during surgery. First it allowed us to refine the theorical nomenclature previously proposed for MRONJ (Odet et al., 2021). Second it assisted us in the practical aspects of our clinical study (Odet et al., 2022).

Of the four techniques evaluated, only two proved to be useful in MRONJ surgery: implantation with complete coverage and implantation with partial coverage. In both techniques, the hAM was very stable as it adhered to the bone and did not move when placing sutures above it. The common aspect of these the two techniques was that wet hAM was buried between the bone and mucosa, which increased its stability.

In contrast, there was no burying of hAM in the apposition technique. Compared to the initial nomenclature, the hAM was not sutured due to its fragility. Thus, wet hAM applied without burying on mucosa/bone nor suturing was unstable and the instability increased with suturing of the gingiva above it. Similarly, the absence of burying in the covering graft technique made hAM unstable on mucosa/bone. In this last technique, suturing of hAM to the gingiva was the hardest part. Suturing of hAM to the mucosa shifted the allograft from the surgical site and made it fold upon itself. This makes hAM unsuitable for the suturing performed during oral surgery. Indeed, the dimensions of the suture material usually range from 3/0 (largest) to 6/0 (thinnest). These types of sutures lacerated the hAM because it is relatively thin. This is in contrast with hAM use in ophthalmology where the allograft is always sutured with smaller suture material—10/0 nylon or 8/10 to 10/0 VICRYL or PROLENE sutures (Sippel et al., 2001)—which produce less cracking of the hAM.

Odet’s review of literature distinguished two types of hAM application in the oral cavity (Odet et al., 2021). First, hAM could be used as an “implanted graft material” in periodontology and implantology. In these cases, the procedure was similar to our “implantation with complete coverage” technique, as the hAM was completely covered by the mucosa. Second, hAM was used as a “covering graft material” in mucosal defects of the oral cavity or in mandibular vestibuloplasties. In these cases, hAM was either directly sutured to the adjacent mucosa or simply applied on the defect to be filled and secured by any means (splints, sutured gauze, etc.) (Samandari et al., 2004; Arai et al., 2012; Amemiya et al., 2015). Here, this second type of surgery involves suturing of hAM which makes it unsuitable to MRONJ.

This technical note supplements the existing literature (Dua and Azuara-Blanco, 1999; Letko et al., 2001; John, 2003) and provides information on how to handle cryopreserved hAM in MRONJ. Detailed and novel illustrations are provided to assist maxillofacial and oral surgeons who want to use hAM in this application.

## Conclusion

This technical note showed that hAM implantation with complete or partial coverage techniques is the preferred choice in an *ex vivo* MRONJ model. Directly suturing to the adjacent mucosa is hardly feasible because of the suture size and the relative thinness of hAM. In oral surgery, cryopreserved hAM has adequate adherence to both bone and mucosa and good stability once applied. This technical note also describes tips and tricks on hAM handling and provides practical illustrations to assist maxillofacial and oral surgeons in hAM application.

## Data Availability

The original contributions presented in the study are included in the article/supplementary materials, further inquiries can be directed to the corresponding author.
